# Effects of Laser Shock Peening on Microstructure and Properties of Ti–6Al–4V Titanium Alloy Fabricated via Selective Laser Melting

**DOI:** 10.3390/ma13153261

**Published:** 2020-07-23

**Authors:** Liang Lan, Ruyi Xin, Xinyuan Jin, Shuang Gao, Bo He, Yonghua Rong, Na Min

**Affiliations:** 1 School of Material Engineering, Shanghai University of Engineering Science, Shanghai 201620, China; lanliang@sues.edu.cn (L.L.); xry692@126.com (R.X.); jinxinyuan1995@163.com (X.J.); gaoshuang_alloy@163.com (S.G.); 2 Research Center of High-temperature Alloy Precision Forming, Shanghai University of Engineering Science, Shanghai 201620, China; 3 School of Materials Science and Engineering, Shanghai Jiao Tong University, Shanghai 200240, China; yhrong@sjtu.edu.cn; 4 Key Laboratory for Microstructures, School of Materials Science and Engineering, Shanghai University, Shanghai 200444, China; minnacy@shu.edu.cn

**Keywords:** laser shock peening, selective laser melting, titanium alloy, microstructure, residual stress, mechanical properties

## Abstract

Laser shock peening (LSP) is an innovative surface treatment process with the potential to change surface microstructure and improve mechanical properties of additively manufactured (AM) parts. In this paper, the influences of LSP on the microstructure and properties of Ti–6Al–4V (Ti64) titanium alloy fabricated via selective laser melting (SLM), as an attractive AM method, were investigated. The microstructural evolution, residual stress distribution and mechanical properties of SLM-built Ti64 samples were characterized before and after LSP. Results show that the SLM sample was composed of single hcp α’ phase, which deviates from equilibrium microstructure at room temperature: α + β phases. The LSP significantly refines the grains of α’ phase and produces compressive residual stress (CRS) of maximum magnitude up to −180 MPa with a depth of 250 μm. Grain refinement of α’ phase is attributed to the complex interaction of dislocations and the intersection of deformation twinning subjected to LSP treatment. The main mechanism of strength and micro-hardness enhancement via LSP is ascribed to the effects of CRS and α’ phase grain refinement.

## 1. Introduction

Ti–6Al–4V (Ti64) alloy is a low density, α + β dual-phase titanium alloy, which exhibits high strength, excellent corrosion resistance and bio-compatibility, and is applied in the aerospace, biomedical and automotive industries [1,2]. However, Ti64 titanium alloy is difficult to cast and machine via traditional methods owing to its inherent properties, such as low thermal conductivity and high chemical reactivity [3]. Additive manufacturing (AM), also known as 3D printing, is considered an innovative manufacturing technology in the industry [4,5,6]. Specifically, selective laser melting (SLM) has become a popular AM method for manufacturing with Ti64 alloy. SLM is based on an incremental layer-by-layer technology, which is able to produce dense, net-shaped complex metallic parts. 

However, the unique conditions in the SLM process lead to coarse grain size and tensile residual stress (TRS) [7,8]. To solve these problems, attempts to optimize the processing parameters of SLM have been investigated extensively. On the other hand, post-processing of titanium alloys has been investigated. Vrancken et al. [9] obtained optimal mechanical properties of SLM-manufactured Ti64 alloy via heat treatment. Colegrove et al. [10] adopted high-pressure rolling to decrease TRS and refine the microstructure of wire arc AM parts. Leuders et al. [11] reported that the hybrid method of combining shot peening (SP) and hot isostatic pressing was a feasible post-treatment process for SLM-manufactured Ti64 alloy, effectively eliminating process-induced defects. Thus, it is reasonably believed that post-treatment technology plays an important part in increasing the mechanical properties of AM components. 

Laser shock peening (LSP) is an innovative post-processing technology, which adopts a high power laser with short pulses to apply on the material surface to produce compressive residual stress (CRS) [12]. LSP offers many advantages compared to traditional SP. For example, the process is able to increase CRS accompanied by larger depth in the near-surface layer without compromising the surface roughness. In addition, enhancements in tensile and fatigue strength have previously been reported via LSP treatment. Jia et al. [13] reported that the improvement in fatigue property of Ti834 alloy was mainly attributed to the introduction of a larger depth of CRS in the surface layer. Irizalp et al. [14] found that 6061-T6 aluminum alloy with LSP had higher strength and ductility than that before LSP, which was ascribed to nanocrystalline, twins and stacking faults generated after LSP. Yang et al. [15] showed that the originally coarse grains were dramatically refined in LSP-treated TC17 alloy. They pointed out that the high strain rate (10^7^ s^−1^) generated via LSP accompanied by the adiabatic temperature increase, which gives rise to rotation dynamic recrystallization proved with quantitative calculation of recrystallization kinetics. Recently, some studies also reported on the hybrid technology combining AM metallic parts and LSP. Kalenticsa et al. [16] found that LSP elevated the microhardness of SLM-built 316 L stainless steel in the near-surface layer, but it did not change the grain size. Guo et al. [17] reported that the tensile strength of Ti64 alloy parts constructed via wire arc AM was improved with LSP. Jin et al. [18] showed that the fatigue strength of the Ti64 alloy manufactured using electron beam melting (EBM) increased by ~17% after being subjected to LSP. However, some important issues, such as stress state, surface roughness and microstructure evolution and their effect on mechanical properties of AM metallic parts treated via LSP, are still pending. Therefore, the influences of LSP on the microstructure and properties of AM Ti64 components deserve thorough investigation, due to the unique conditions during the various AM processes.

In the present work, we investigated comprehensively the influences of LSP on the microstructure and mechanical properties of Ti64 alloy fabricated via SLM. We focus on the differences in microstructural evolution among SLM Ti64, EBM Ti64 alloy and wire arc AM Ti64 alloy with LSP because they possess different stress states under the various AM processes. The microstructure evolution, residual stress, micro-hardness and tensile strength of SLM-manufactured Ti64 alloy before and after LSP were analyzed. The enhancement mechanism of tensile strength and micro-hardness subjected to LSP was discussed. This study will promote the applications of the SLM-manufactured titanium alloy parts.

## 2. Experimental Procedures

**Figure 1:**
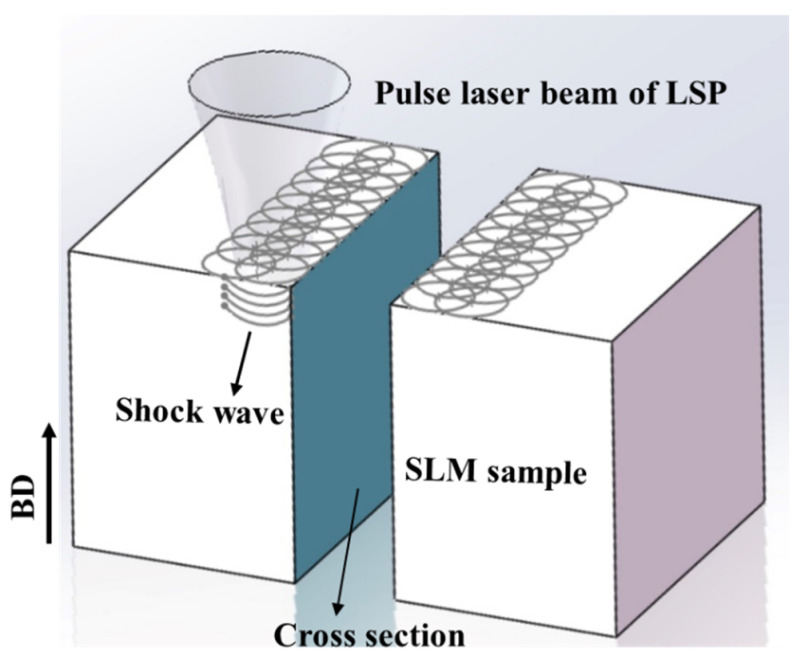
Schematic of the laser shock peening (LSP) principle for a selective laser melting (SLM) sample.

SLM manufacturing was conducted on an EOS M280 machine with gas-atomized Ti64 powder. Table 1 shows the composition of Ti64 powder used in the present study. The SLM machine employs a strategy of short line scanning with a rotation angle of 67° between layers with thicknesses of 30 μm. A laser beam with a spot size of ~100 μm and a hatch spacing of 140 μm was used. The laser power was 280 W, and the scanning speed was 1200 mm/s. All the specimens were fabricated on the pure titanium substrate with dimensions of 250 × 250 × 15 mm^3^. During SLM, an argon atmosphere was used, and the O_2_ concentration was in a range of 0.05–0.1 vol.%. After the samples were produced via SLM, LSP treatment was applied on the surface of the formed samples. Samples with a size of 10 × 10 × 6 mm^3^ were cut and conducted by one side LSP for microstructure observation. During LSP, an Nd:YAG laser with a wavelength of 1064 nm, and a laser power intensity of 11.89 GW·cm^−2^ was used. The pulse duration was 12 ns, and the diameter of the laser beam was set to 2.5 mm. The overlapping rate between the two laser paths was 25% with a zigzag manner. Figure 1 presents a schematic of the LSP principle for an SLM-manufactured Ti64 sample.

Samples were ground on SiC sandpapers, polished with a diamond slurry, and then etched in a mixture of hydrofluoric acid (5 mL), nitric acid (25 mL) and water (50 mL). The microstructure and phase composition of the specimens were characterized using an optical microscope (OM), X-ray diffraction (XRD) and electron backscattering diffraction (EBSD). A transmission electron microscope (TEM) was adopted to analyze the cross-sectional microstructure evolution of SLM-built specimens before and after laser peening. The residual stresses of specimens before and after LSP were conducted using an XRD test with the sin^2^ψ method. A Proto LXRD2000 diffractometer (Proto, Windsor, ON, Canada) with Cu Kα (λ = 1.541838 Å) was employed. Lattice strain measurement was conducted at the {2 1 3}-planes of the hexagonal a-phase. The X-ray tube voltage and current were 30 kV and 25 mA, respectively. To measure the residual stress with the depth, the sample was removed layer by layer from the surface using electro-polishing in steps of 50 μm, and another measurement was conducted on the exposed surface.

The density of the SLM samples was measured as 99.15% using the Archimedes’ method. The micro-hardness with depth was measured using a micro-hardness tester. Each measurement was conducted five times. A tensile dog-bone specimen was machined from 70 × 15 × 20 mm^3^ blocks via wire-electrode cutting along the perpendicular to building direction (BD). The gauge cross-section was 4 (width) × 2 (thickness) mm^2^ with a gauge length of 15 mm. The tensile tests for as-built specimens before and after laser peening was performed using a strain rate of 5.5 × 10^−4^/s. Three specimens were conducted for each condition. LSP was performed on two sides covering the gauge length region. 

## 3. Results

### 3.1. Microstructure Characterization

**Figure 2:**
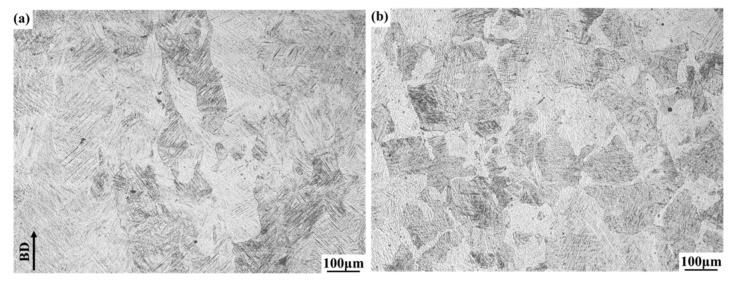
Optical microscope (OM) of SLM-fabricated Ti64 samples (**a**) side (**b**) top view.

Figure 2 presents the OM images of the microstructures of the Ti64 samples manufactured via SLM. Few voids were found, but no obvious cracks were observed in the Ti64 samples. Figure 2a displays the original β columnar grains parallel to the BD, and the top view shows equiaxed grains parallel to the sample surface (Figure 2b). The OM observation shows that a lamellar microstructure was generated in SLM. 

**Figure 3:**
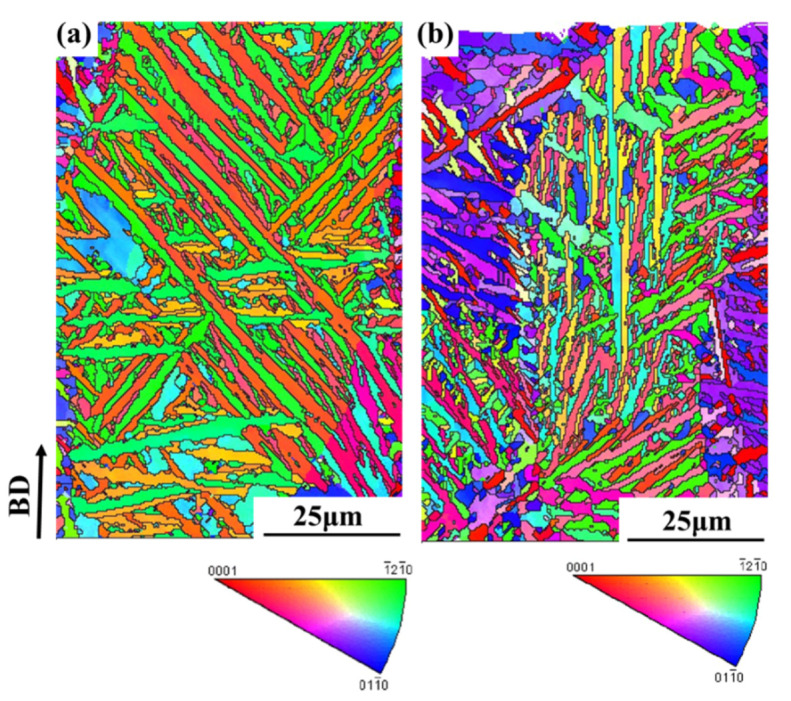
Electron backscattering diffraction (EBSD) maps of the cross-section for SLM-fabricated Ti64 samples before (**a**) and after (**b**) LSP.

Figure 3 shows the EBSD maps of the cross-section of SLM-manufactured Ti64 samples before and after LSP, respectively. It is clear that the SLM-built Ti64 sample exhibits many long and straight laths, and they are identified as hexagonal close-packed (hcp) phase, and no body centered cubic (bcc) β phase exists, as shown in Figure 3a, which deviates from equilibrium microstructure at room temperature: α + β phases. Such a phenomenon was analyzed as follows. During SLM, very rapid cooling in the orders of thousands of degrees per second for each layer building results in the diffusionless martensitic transformation of bcc β → hcp α’ in the SLM-manufactured Ti64 sample [19]. Therefore, the hcp phase measured by XRD is metastable α´ phase, rather than stable hcp α phase. Their orientations mainly focus on or near [0001] and [$\bar{1}$2$\bar{1}$0] of the hcp phase, and a few orientations are on or near [01$\bar{1}$0], but long and straight laths markedly decrease in the sample treated by LSP (Figure 3b), indicating that the hcp phase is refined. Meanwhile, their orientations also change, that is, [01$\bar{1}$0] significantly increases accompanied by the decrease of [0001] after LSP. According to Zhu et al. [20], the grain refinement and orientation change of α´ phase are attributed to the plastic deformation during LSP.

**Figure 4:**
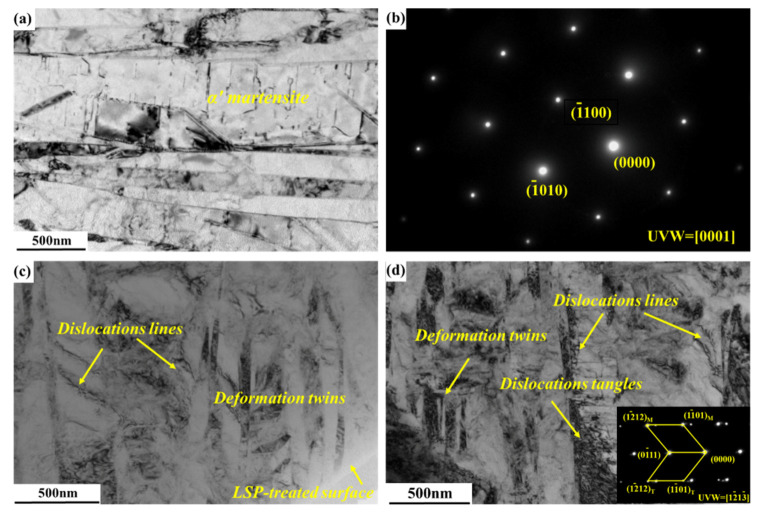
TEM images of SLM Ti64 specimens (**a**,**b**) before and (**c**,**d**) after LSP. (**a**) Coarse α’ martensite, (**b**) selective area diffraction pattern (SADP) of hcp α’ phase, (**c**) refined α’ martensite and (**d**) dislocations, deformation twins and the corresponding SADP.

Figure 4 further demonstrates TEM images of SLM-fabricated Ti64 specimens before and after laser peening. Figure 4a exhibits a coarse martensite structure in the SLM-fabricated Ti64 specimen, which was identified as hcp α’ phase by selective area diffraction pattern (SADP), as shown in Figure 4b. In the coarse α’ martensite structure, there is a very low density of dislocations, in which single dislocation is observed. Figure 4c shows a refined α’ martensite structure in the SLM-fabricated Ti64 specimen subjected to LSP. The deformed twins of α’ phase oriented in two directions were observed after LSP in Figure 4c,d, which was verified by the SADP. Moreover, there are numerous dislocation structures, including dislocation lines and dislocation tangles concentrating in the α’ martensites.

### 3.2. Residual Stress Distribution

**Figure 5:**
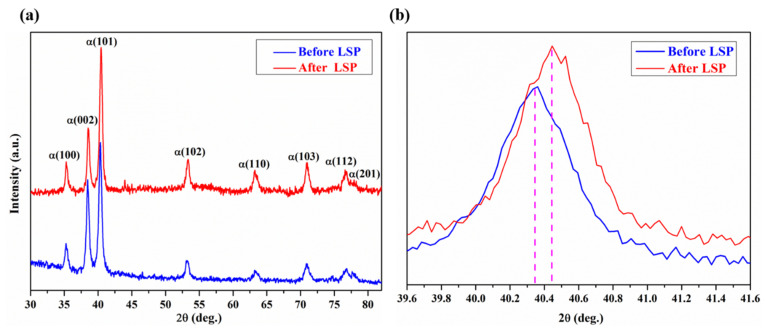
(**a**) XRD of the surface layer in SLM Ti64 samples before and after LSP; (**b**) diffraction peaks shifting of α (101).

XRD measurement was performed on the surface layer of as-built Ti64 samples before and after LSP. From Figure 5, XRD patterns show a single hcp phase, which is consistent with EBSD, and thus the hcp phase is α’ martensite. From Figure 5a, LSP does not produce a new phase, but the diffraction peaks shift into a higher angle (Figure 5b). The full width at half maximum of the diffraction peak increases, which may be a result of grain refinement on the surface layer of the SLM-built Ti64 sample via LSP. 

**Figure 6:**
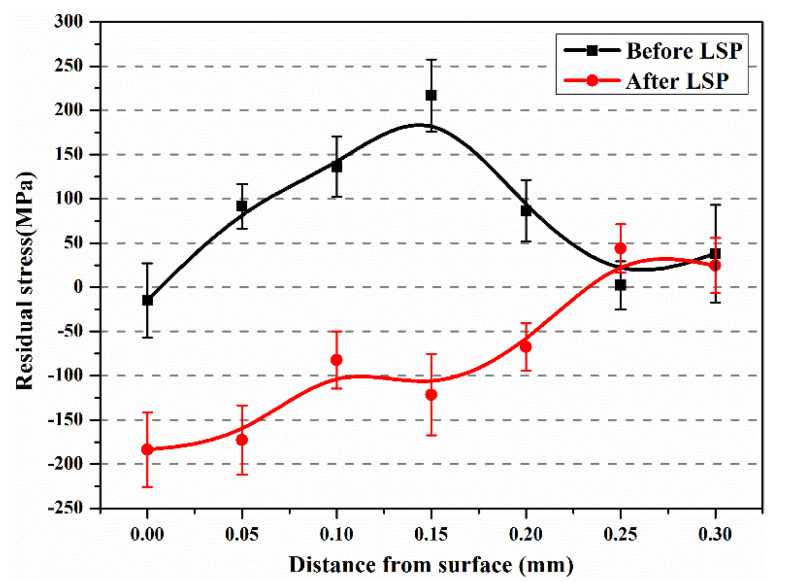
Residual stress of SLM-fabricated Ti64 samples before and after LSP.

Figure 6 displays the residual stress distribution of the as-built Ti64 sample before and after laser peening. It was observed that TRS was generated on the surface of the as-built Ti64 specimen before LSP. The TRS is an outcome of competition between thermal stress and phase transformation stress, in which the former is favorable for compressive stress formation, and the latter is favorable for the tensile stress formation [21]. Thus, it is reasonable to infer that during SLM the tensile stress produced by bcc β → hcp α´ is greater than the compressive stress produced by the high-temperature gradients, which results in the maximum tensile stress of 216 MPa at a depth of 150 μm. Kalentics et al. [22] also reported that TRS reached 342 MPa in the SLM-built 316 L stainless steel. After LSP, CRS was generated at the subsurface, and the maximum value was about −180 MPa. With increasing the depth from the surface, the compressive stress reduced and converted into tensile stress. Usually, the depth of CRS was applied to present the plastically affected depth. It can be found that the plastically affected depth was ~250 μm, which is lower than the affected depth of CRS in the EBM-built Ti64 sample with LSP [23]. 

### 3.3. Mechanical Properties

**Figure 7:**
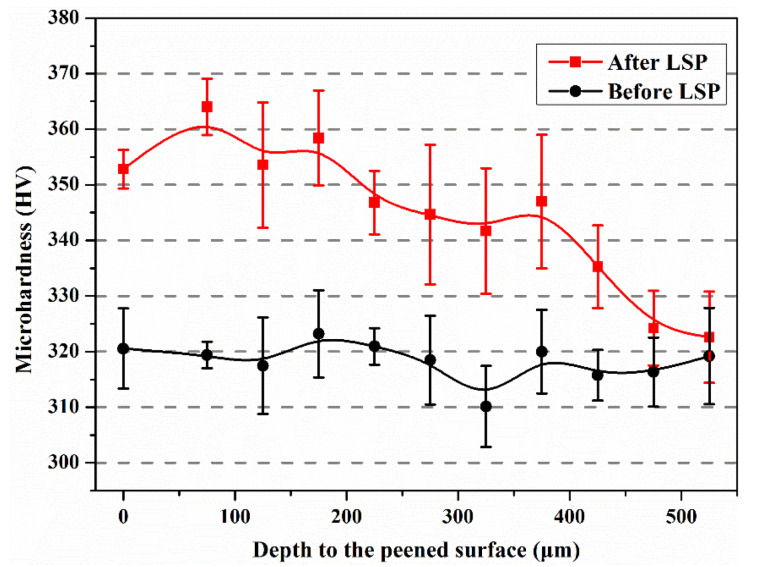
Microhardness of SLM-built Ti64 samples before and after LSP with depth.

The in-depth micro-hardness distribution of the SLM-built Ti64 sample before and after laser peening is shown in Figure 7. The average micro-hardness of the SLM-built Ti64 sample before LSP was ~318 HV. After laser peening, the average micro-hardness increased to ~353 HV, which was ~10% higher than that before LSP. From Figure 7, the micro-hardness of SLM-produced Ti64 alloy decreased with increasing the depth away from the surface. The thickness of the hardening layer was ~475 μm. The results indicated that the micro-hardness of SLM-built Ti64 samples was improved via LSP. Peyre et al. [24] showed that LSP was a suitable process to acquire higher micro-hardness and larger depth of the affected layer. However, it is noted that the thickness of the hardening layer was 225 μm higher than that of the affected layer of residual stress. According to Multigner et al. [25], it is difficult for residual stress to transfer along the depth direction owing to low strain hardening exponent, even though twinning and dislocation are generated in the Ti64 sample. Moreover, O_2_ is involved in the SLM process of Ti64 alloy, leading to grain growth restriction and hence contributing to the hardening effect [26].

**Figure 8:**
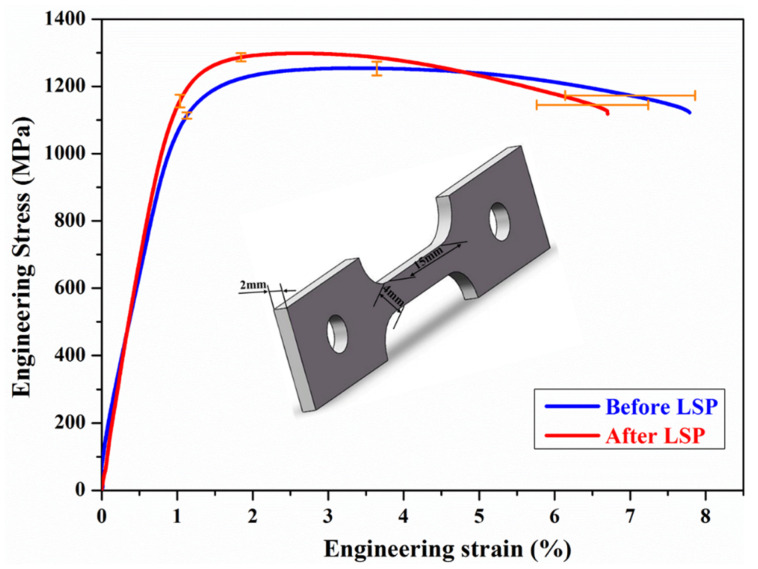
Engineering stress–strain curves of SLM-fabricated Ti64 samples before and after LSP; error bars represent one standard deviation.

The tensile strength of SLM-fabricated Ti64 samples with LSP was investigated. Figure 8 shows the typical engineering stress–strain plots of the samples before and after laser peening. The stress load was applied in normal to the BD. It can be found that the strength of LSP-treated samples surpasses that of as-built samples via SLM. Table 2 shows the yield strength (YS), ultimate tensile strength (UTS) and elongation (El) of samples before and after LSP. It was seen that the UTS and YS of the samples were improved and a slight decrease in El was observed after LSP. The results indicated that LSP could eliminate the negative effects induced via SLM and increase the tensile properties of SLM-fabricated samples. Sun et al. [27] also reported that LSP treatment enhanced the tensile properties of 2319 aluminum alloy formed by wire arc AM. In the present work, CRS affected layer and grain refinement were achieved in the samples, which could be the dominant factor of tensile strength enhancement.

## 4. Discussions

### 4.1. Evolution of the Microstructural under LSP

According to the TEM observation results in Figure 4, the refined grains in the SLM-fabricated Ti64 specimen were achieved after being subjected to LSP. The near-surface microstructure of the SLM-built Ti64 specimen after LSP comprised of deformation twins and high-density dislocations. The formation of twins can be explained as follows. As is well known, the hcp α’ phase has low stacking fault energy and only three slip systems, but according to the Von-Mises rule, five independent slip systems are necessary to coordinate plastic deformation [28,29]. Thus, when the dislocation slip is hindered, twinning can coordinate plastic deformation since the formation of deformed twins accompanied by changes in crystallographic orientation is conducive to compatible deformation among grains. 

During LSP, α’ phase grain refinement is dominated by dislocation slip and deformed twinning. At first, under laser-induced shockwave, plastic deformation is produced in the original grains of hcp α’ phase in SLM-fabricated Ti64 alloy. In the initial stage of deformation, the hcp α’ grains in SLM-built Ti64 alloy deform mainly via dislocation slip during LSP. As the plastic strain accumulates, i.e., the force of laser shockwaves up to the level of critical twinning stress, deformed twins can be generated in the hcp α’ grains [30]. Then the coarse α’ grains are divided via the complex interaction of dislocations and the intersection of deformed twins. As mentioned above, the plastic deformation under the action of laser shockwaves was considered as an adiabatic process, and thus a high temperature was induced via the transforming of mechanical work to heat. Figure 4c,d shows that there are not nano-equiaxed grains in the near-surface layer of LSP-treated samples. It indicates that the adiabatic increased temperature generated by LSP is not enough to produce dynamic recrystallization so that nano-equiaxed grains cannot form in the near-surface layer.

### 4.2. Strengthening Mechanism Induced via LSP

As is well known, laser peening can induce CRS of several hundred MPa. In-depth residual stress distribution of SLM samples in Figure 6 shows that LSP treatment leads to significant improvement in the CRS on the surface. Further, it is clear from Figure 6 that LSP produces a CRS layer with a maximum value of −180 MPa which decreases to zero at a depth of 250 μm. The compressive stress of −180 MPa generated via LSP contributes to pre-existing void closure, retarding crack propagation. In addition, the CRS neutralizes part of the TRS in the tensile specimen. Grain refinement usually contributes to the strength of metallic materials according to the Hall–Petch equation [31]. It is attributed to the influence of grain boundaries which can prevent dislocation motion and raise the difficulty for dislocations traversing grain boundaries as reported by Schino et al. [32]. From Figure 4c, the refined α’ martensite structure was observed in the SLM-fabricated specimen subjected to laser peening. The increase in strength of the specimen results from the grain refinement. Therefore, the strengthening mechanism via LSP was ascribed to the effects of CRS and α’ phase grain refinement.

### 4.3. Effect of Refined Grains on Micro-Hardness

As shown in Figure 7, in-depth micro-hardness distribution of the SLM-fabricated Ti64 sample before and after laser peening was characterized. The micro-hardness of the SLM-fabricated Ti64 sample increased from 318 HV to 353 HV after being subjected to LSP, namely, an increase of 10%. The increase of micro-hardness is attributed to the increase of dislocation density and grain refinement by LSP. The influence of grain refinement on micro-hardness was evaluated according to the well-established Hall–Petch equation [31], which reflects the relationship between the micro-hardness (*H*_v_) and the average grain size (*d*) of the materials:

(1) $$H_{v}=H_{0}+\frac{K_{h}}{\sqrt{d}}$$

where *H*_0_ and *K*_h_ are the materials constant. In Equation (1), the *H*_0_ (347) and *K_h_* (904) were calculated based on the titanium alloy data of Tong et al. [33] in which the micro-hardness is increased by ~8% when the grain size is refined from 450 to 150 nm, while the micro-hardness measured is raised by ~10%, which implies that the rest of the increase in micro-hardness is ascribed to the increase in dislocation density.

## 5. Conclusions

The influences of LSP on the microstructure and mechanic properties of SLM-built Ti64 samples were investigated. Microstructural evolution, residual distribution and mechanical properties of as-built Ti64 samples were characterized via comparing before with after LSP. The main conclusions are described as follows.

(1) The EBSD map of SLM-built Ti64 sample exhibits coarse hcp α’ martensite laths, and no β phase exists, which deviates from equilibrium microstructure at room temperature: hcp α + bcc β.
(2) The LSP treatment produces CRS of maximum magnitude up to −180 MPa, and the depth of the CRS layer reaches 250 μm.
(3) Grain refinement of α’ phase is ascribed to the complex interaction of dislocations and the intersection of deformation twinning after LSP.
(4) The tensile strength and micro-hardness of SLM-fabricated Ti64 samples was improved after subjecting to LSP, which was attributed to the grain refinement of α’ phase and CRS.

## Figures and Tables

**Table 1:** Chemical composition of Ti64 powder.

Composition	Al	V	Fe	N	O	H	Ti
Mass fraction/%	6.2	3.89	0.17	0.002	0.091	0.002	Bal.

**Table 2:** Tensile properties of SLM-fabricated Ti64 samples before and after LSP.

Ti64 Samples	Before LSP	After LSP
UTS/MPa	1253.3 ± 20.54	1286.7 ± 12.48
YS/MPa	1113.3 ± 9.43	1156.7 ± 18.86
El/%	7.0 ± 0.86	6.5 ± 0.74

## References

[1] Manero J.M., Gil F.J., Planell J.A. (2000). Deformation Mechanisms of Ti-6Al-4V Alloy with a martensitic microstructure subjected to oligocyclic fatigue. Acta Mater..

[2] Voisin T., Calta N.P., Khairallah S.A., Forien J.-B., Balogh L., Cunningham R.W., Rollett A.D., Wang Y.M. (2018). Defects-dictated tensile properties of selective laser melted Ti-6Al-4V. Mater. Des..

[3] Qian M., Xu W., Brandt M., Tang H.P. (2016). Additive manufacturing and post processing of Ti-6Al-4V for superior mechanical properties. MRS Bull..

[4] Wang Y.M., Voisin T., McKeown J., Ye J., Calta N.P., Li Z., Zeng Z., Zhang Y., Chen W., Roehling T.T. (2018). Additively manufactured hierarchical stainless steels with high strength and ductility. Nat. Mater..

[5] Zhang D., Qiu D., Gibson M.A., Zheng Y., Fraser H.L., StJohn D.H., Easton M.A. (2019). Additive manufacturing of ultrafine-grained high-strength titanium alloys. Nature.

[6] Chen C., Xie Y., Yan X., Yin S., Fukanuma H., Huang R., Zhao R., Wang J., Ren Z., Liu M. (2019). Effect of Hot Isostatic Pressing (HIP) on microstructure and mechanical properties of Ti-6Al-4V alloy fabricated by cold spray additive manufacturing. Addit. Manuf..

[7] Chen W., Voisin T., Zhang Y., Florien J.-B., Spadaccini C.M., McDowell D.L., Zhu T., Wang Y.M. (2019). Microscale residual stresses in additively manufactured stainless steel. Nat. Commun..

[8] Haar G.M.T., Becker T.H. (2018). Selective laser melting produced Ti-6Al-4V: Post-process heat treatments to achieve superior tensile properties. Materials.

[9] Vrancken B., Thijs L., Kruth J.-P., van Humbeeck J. (2012). Heat treatment of Ti-6Al-4V produced by selective laser melting: Microstructure and mechanical properties. J. Alloy Compd..

[10] Colegrove P.A., Coules H.E., Fairman J., Martina F., Kashoob T., Mamash H., Cozzolino L.D. (2013). Microstructure and residual stress improvement in wire and arc additively manufactured parts through high-pressure rolling. J. Mater. Process. Technol..

[11] Leuders S., Meiners S., Wu L., Taube A., Tröster T., Niendorf T. (2017). structural components manufactured by selective laser melting and investment casting impact of the process route on the damage mechanism under cyclic loading. J. Mater. Process. Technol..

[12] Liao Y., Ye C., Cheng G.J. (2016). A review: Warm laser shock peening and related laser processing technique. Opt. Laser Technol..

[13] Jia W., Hong Q., Zhao H., Li L., Han D. (2014). Effect of laser shock peening on the mechanical properties of a near-α titanium alloy. Mater. Sci. Eng..

[14] Irizalp S.G., Saklakoglu N. (2016). High strength and high ductility behavior of 6061-T6 alloy after laser shock processing. Opt. Laser Eng..

[15] Yang Y., Zhang H., Qiao H. (2017). Microstructure characteristics and formation mechanism of TC17 titanium alloy induced by laser shock processing. J. Alloy Compd..

[16] Kalentics N., Huang K., de Seijas M.O.V., Burn A., Romano V., Logé R. (2019). Laser shock peening: A promising tool for tailoring metallic microstructures in selective laser melting. J. Mater. Process. Technol..

[17] Guo W., Sun R., Song B., Zhu Y., Li F., Che Z., Li B., Guo C., Liu L., Peng P. (2018). Laser shock peening of laser additive manufactured Ti6Al4V titanium alloy. Surf. Coatings Technol..

[18] Jin X., Lan L., Gao S., He B., Rong Y. (2020). Effects of laser shock peening on microstructure and fatigue behavior of Ti–6Al–4V alloy fabricated via electron beam melting. Mater. Sci. Eng..

[19] Yang J., Yu H., Yin J., Gao M., Wang Z., Zeng X. (2016). Formation and control of martensite in Ti-6Al-4V alloy produced by selective laser melting. Mater. Des..

[20] Zhu X., Zhou M., Dai Q., Cheng G.J. (2009). Deformation modes in stainless steel during laser shock peening. j. manuf. sci. eng..

[21] Liu Y., Qin S., Zhang J., Wang Y., Rong Y., Zuo X., Chen N. (2017). Influence of transformation plasticity on the distribution of internal stress in three water-quenched cylinders. Met. Mater. Trans..

[22] Kalenticsa N., Boillata E., Peyreb P., Gorny C., Kenel C., Leinenbach C., Jhabvala J., Logé R.E. (2017). 3D laser shock peening—A new method for the 3D control of residual stresses in selective laser melting. Mater. Des..

[23] Lan L., Jin X., Gao S., He B., Rong Y. (2020). Microstructural evolution and stress state related to mechanical properties of electron beam melted Ti–6Al–4V alloy modified by laser shock peening. J. Mater. Sci. Technol..

[24] Peyre P., Fabbro R., Merrien P., Lieurade H. (1996). Laser shock processing of aluminium alloys. application to high cycle fatigue behaviour. Mater. Sci. Eng..

[25] Multigner M., Frutos E., Mera C., Chao J., González-Carrasco J.L. (2009). Interrogations on the sub-surface strain hardening of grit blasted Ti-6Al-4V alloy. Surf. Coatings Technol..

[26] Oh J.-M., Lim J.-W., Lee B.-G., Suh C.-Y., Cho S.-W., Lee S.-W., Choi G.-S. (2010). Grain refinement and hardness increase of titanium via trace element addition. Mater. Trans..

[27] Sun R., Li L., Zhu Y., Guo W., Peng P., Cong B., Sun J., Che Z., Li B., Guo C. (2018). Microstructure, residual stress and tensile properties control of wire-arc additive manufactured 2319 aluminum alloy with laser shock peening. J. Alloy Compd..

[28] Leyens C., Peters M. (2003). Titanium and Titanium Alloys: Fundamentals and Applications.

[29] Bridier F., Villechaise P., Mendez J. (2005). Analysis of the different slip systems activated by tension in a α/β titanium alloy in relation with local crystallographic orientation. Acta Mater..

[30] Meyers M.A., Vöhringer O., Lubarda V. (2001). The Onset of twinning in metals: A constitutive description. Acta Mater..

[31] Yin F., Cheng G.J., Xu R., Zhao K., Li Q., Jian J., Hu S., Sun S., An L., Han Q. (2018). Ultrastrong nanocrystalline stainless steel and its hall-petch relationship in the nanoscale. Scr. Mater..

[32] Di Schino A., Kenny J. (2003). Grain refinement strengthening of a micro-crystalline high nitrogen austenitic stainless steel. Mater. Lett..

[33] Tong Z., Ren X., Ren Y., Dai F., Ye Y., Zhou W., Chen L., Ye Z. (2018). Effect of laser shock peening on microstructure and hot corrosion of TC11 alloy. Surf. Coatings Technol..

